# Supplementary material for: Population genomics of the Anthropocene: urbanization is negatively associated with genome‐wide variation in white‐footed mouse populations
Source: Evol Appl. 2016 Feb 11;9(4):546–64. doi: 10.1111/eva.12357 (PMC4831458; doi:10.1111/eva.12357)
Supplement: Supplementary file 1 — Table S1. Correlation coefficients calculated between % impervious surface and human population sizes calculated at buffers around study sites of 500 m, 1 km, 1.5 km, and 2 km. [file EVA-9-546-s001.docx]

**Table S1.** Pearson correlation coefficients calculated between % impervious surface (imprv) and human population sizes (pop) calculated at buffers around study sites of 500 m, 1 km, 1.5 km, and 2 km. Correlations of *r* > 0.90 are highlighted in bold.

|  | **pop500m** | **pop1km** | **pop1.5km** | **pop2km** | **imprv500m** | **imprv1km** | **imprv1.5km** |
| --- | --- | --- | --- | --- | --- | --- | --- |
| **pop500m** | 1.000 |  |  |  |  |  |  |
| **pop1km** | **0.939** | 1.000 |  |  |  |  |  |
| **pop1.5km** | **0.931** | **0.993** | 1.000 |  |  |  |  |
| **pop2km** | 0.891 | **0.975** | **0.990** | 1.000 |  |  |  |
| **imprv500m** | 0.824 | 0.884 | 0.900 | **0.915** | 1.000 |  |  |
| **imprv1km** | 0.652 | 0.797 | 0.820 | 0.866 | **0.909** | 1.000 |  |
| **imprv1.5km** | 0.623 | 0.774 | 0.805 | 0.860 | 0.882 | **0.992** | 1.000 |
| **imprv2km** | 0.563 | 0.722 | 0.758 | 0.824 | 0.843 | **0.979** | **0.995** |
