# Supplementary material for: Population genomics of the Anthropocene: urbanization is negatively associated with genome‐wide variation in white‐footed mouse populations
Source: Evol Appl. 2016 Feb 11;9(4):546–64. doi: 10.1111/eva.12357 (PMC4831458; doi:10.1111/eva.12357)
Supplement: Supplementary file 2 — Table S2. Summary population genomic statistics calculated for all nucleotide positions (variant and fixed). [file EVA-9-546-s002.docx]

**Table S2**. Summary genetic diversity statistics calculated by STACKS for all nucleotide positions. *N* = average number of individuals genotyped at each locus; *Sites* = number of polymorphic nucleotide sites across the dataset; *%Poly* = percentage of polymorphic loci; *Private* = number of variable sites unique to each population; *P* = average frequency of the major allele; *H_obs_ =* average observed heterozygosity per locus; *π* = average nucleotide diversity.

| **Site** | **Type** | **N** | **Sites** | **%Poly** | **P** | **H_obs_** | **π** |
| --- | --- | --- | --- | --- | --- | --- | --- |
| AP | City | 10.53 | 1,462,036 | 0.00388 | 0.9994 | 0.0008 | 0.0009 |
| CP | City | 6.71 | 1,379,677 | 0.00321 | 0.9993 | 0.001 | 0.001 |
| FM | City | 7.81 | 1,457,433 | 0.00229 | 0.9995 | 0.0006 | 0.0007 |
| FP | City | 5.41 | 1,466,647 | 0.00224 | 0.9995 | 0.0007 | 0.0008 |
| FT | City | 3.70 | 1,463,962 | 0.00235 | 0.9995 | 0.0008 | 0.0009 |
| IP | City | 5.30 | 1,464,152 | 0.00304 | 0.9994 | 0.0008 | 0.0009 |
| JB | City | 4.35 | 1,451,101 | 0.00230 | 0.9995 | 0.0008 | 0.0008 |
| KP | City | 7.85 | 1,463,479 | 0.00278 | 0.9995 | 0.0007 | 0.0008 |
| NYBG | City | 8.12 | 1,244,138 | 0.00321 | 0.9992 | 0.0012 | 0.0011 |
| PB | City | 9.32 | 1,455,791 | 0.00414 | 0.999 | 0.0017 | 0.0015 |
| RR | City | 7.73 | 1,441,686 | 0.00209 | 0.9996 | 0.0005 | 0.0007 |
| VC | City | 5.60 | 1,467,127 | 0.00350 | 0.9993 | 0.0012 | 0.0012 |
| CPV | Suburb | 6.21 | 1,448,118 | 0.00383 | 0.999 | 0.0017 | 0.0014 |
| LCC | Suburb | 10.74 | 1,444,843 | 0.00450 | 0.999 | 0.0016 | 0.0014 |
| MRG | Suburb | 5.28 | 1,460,207 | 0.00371 | 0.999 | 0.0017 | 0.0015 |
| SW | Suburb | 5.74 | 1,446,488 | 0.00303 | 0.9994 | 0.0008 | 0.0009 |
| CFP | Rural | 7.16 | 1,458,384 | 0.00380 | 0.9991 | 0.0014 | 0.0012 |
| CIE | Rural | 5.70 | 1,416,348 | 0.00300 | 0.9995 | 0.0008 | 0.0009 |
| HIP | Rural | 7.09 | 1,466,249 | 0.00416 | 0.9991 | 0.0015 | 0.0013 |
| HP | Rural | 7.22 | 1,457,226 | 0.00452 | 0.9991 | 0.0014 | 0.0014 |
| MH | Rural | 7.10 | 1,467,503 | 0.00432 | 0.9989 | 0.0018 | 0.0015 |
| MR | Rural | 6.35 | 1,462,035 | 0.00295 | 0.9995 | 0.0007 | 0.0009 |
| WW | Rural | 6.78 | 1,439,770 | 0.00327 | 0.9992 | 0.0013 | 0.0011 |
| Mean |  | 6.86 | 1,442,800 | 0.00331 | 0.9993 | 0.0011 | 0.0011 |
