# Supplementary figures and images for: Population genomics of the Anthropocene: urbanization is negatively associated with genome‐wide variation in white‐footed mouse populations
Source: Evol Appl. 2016 Feb 11;9(4):546–64. doi: 10.1111/eva.12357 (PMC4831458; doi:10.1111/eva.12357)

# a-score optimisation - spline interpolation

Optimal number of PCs: 23

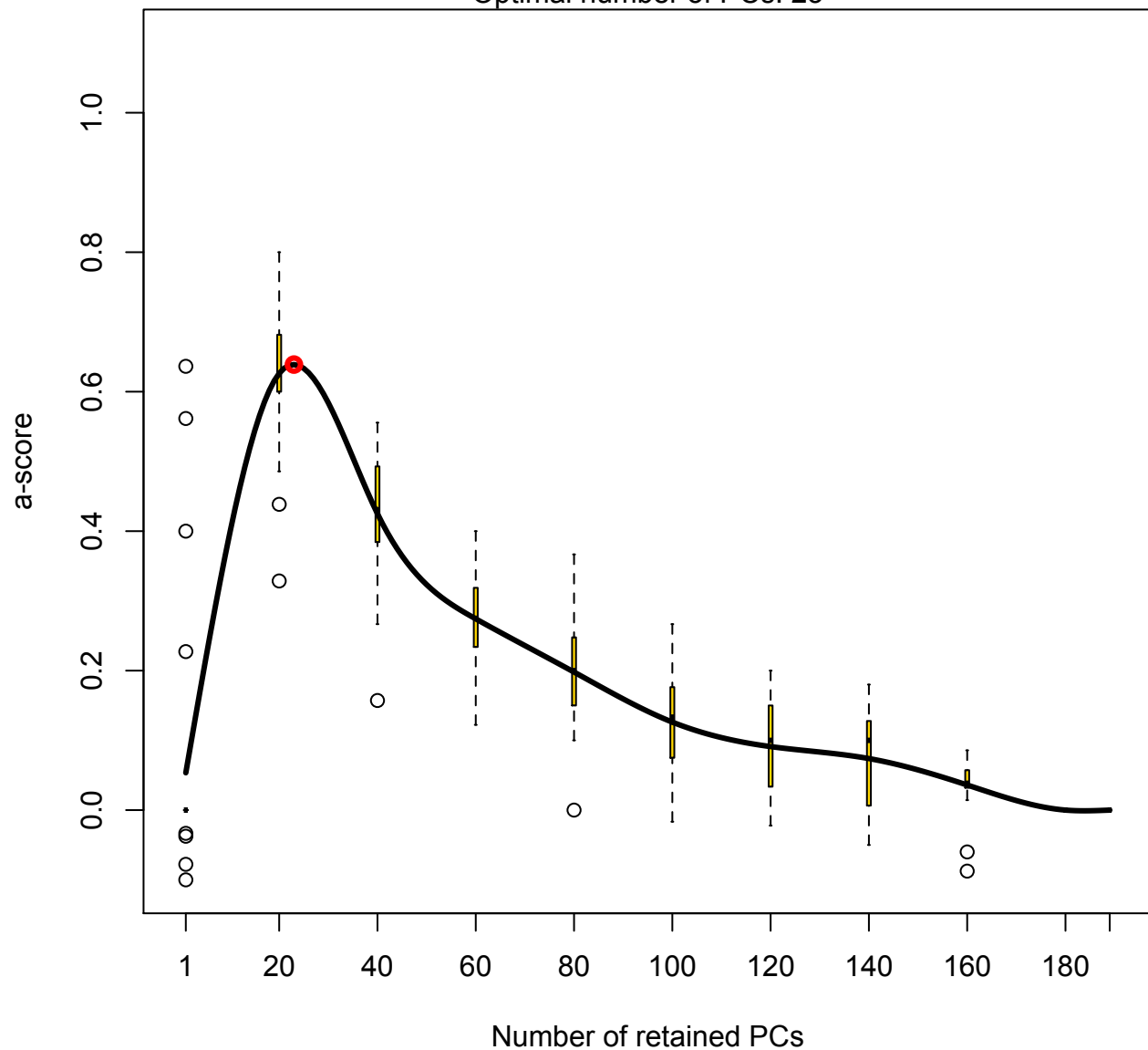

Supplement: Supplementary file 4 — Figure S1. Cross‐validation (i.e., a‐score optimization) to identify the optimal number of principal components to retain for DAPC without overfitting4. [file EVA-9-546-s004.pdf]

membership probability

1.0  
0.8  
0.6  
0.4  
0.2  
0.0

individuals

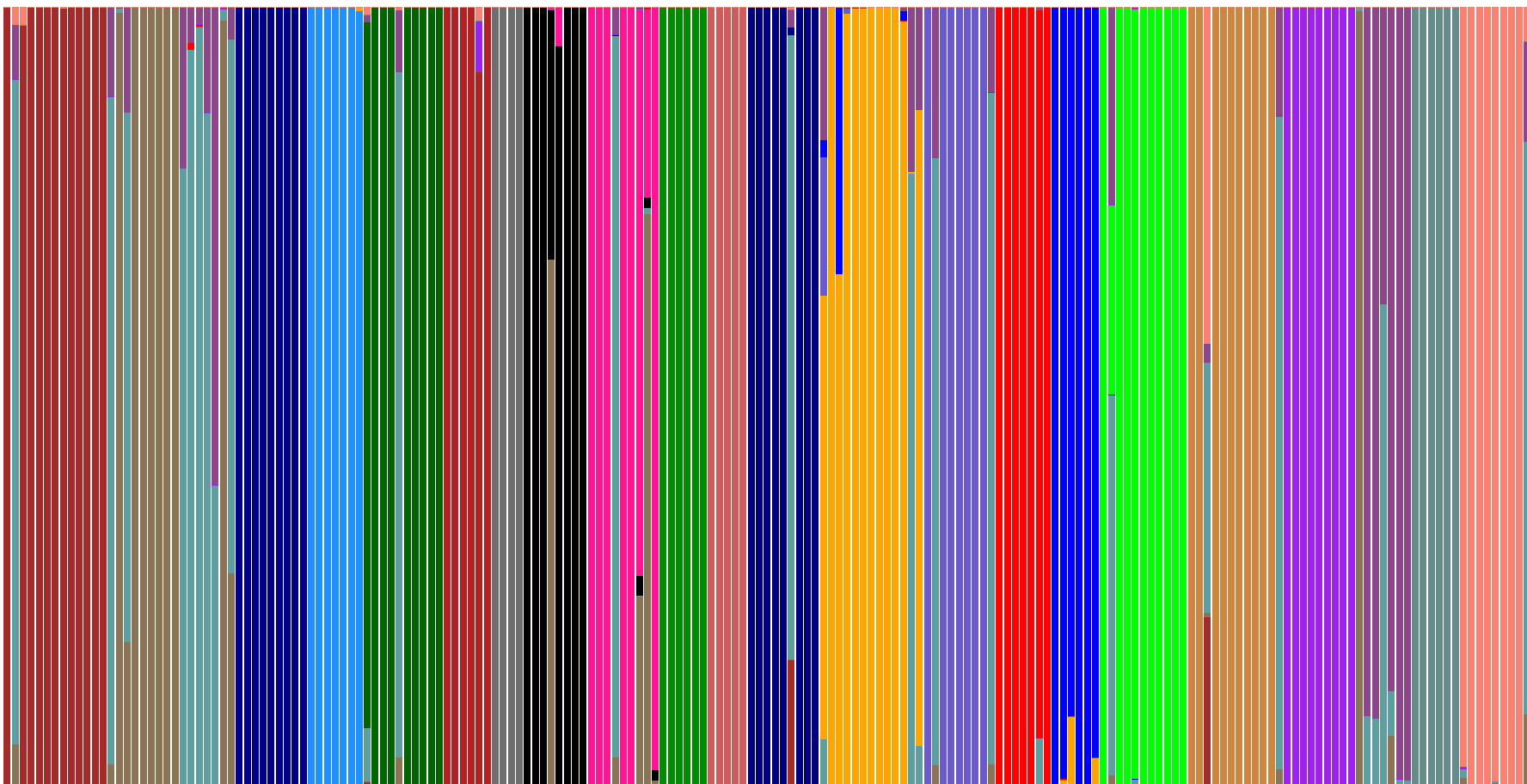

Supplement: Supplementary file 5 — Figure S2. Compoplot/bar plot result from DAPC analysis on all 23 populations. [file EVA-9-546-s005.pdf]

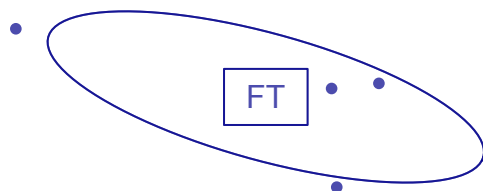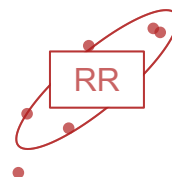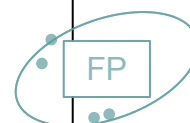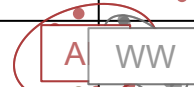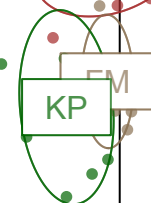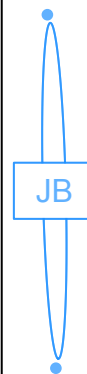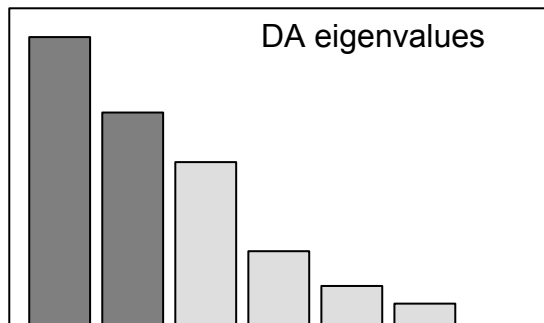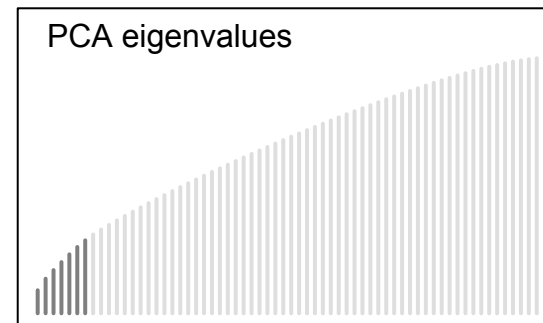

Supplement: Supplementary file 6 — Figure S3. Scatterplot of first two discriminant functions from DAPC for populations on Long Island. [file EVA-9-546-s006.pdf]

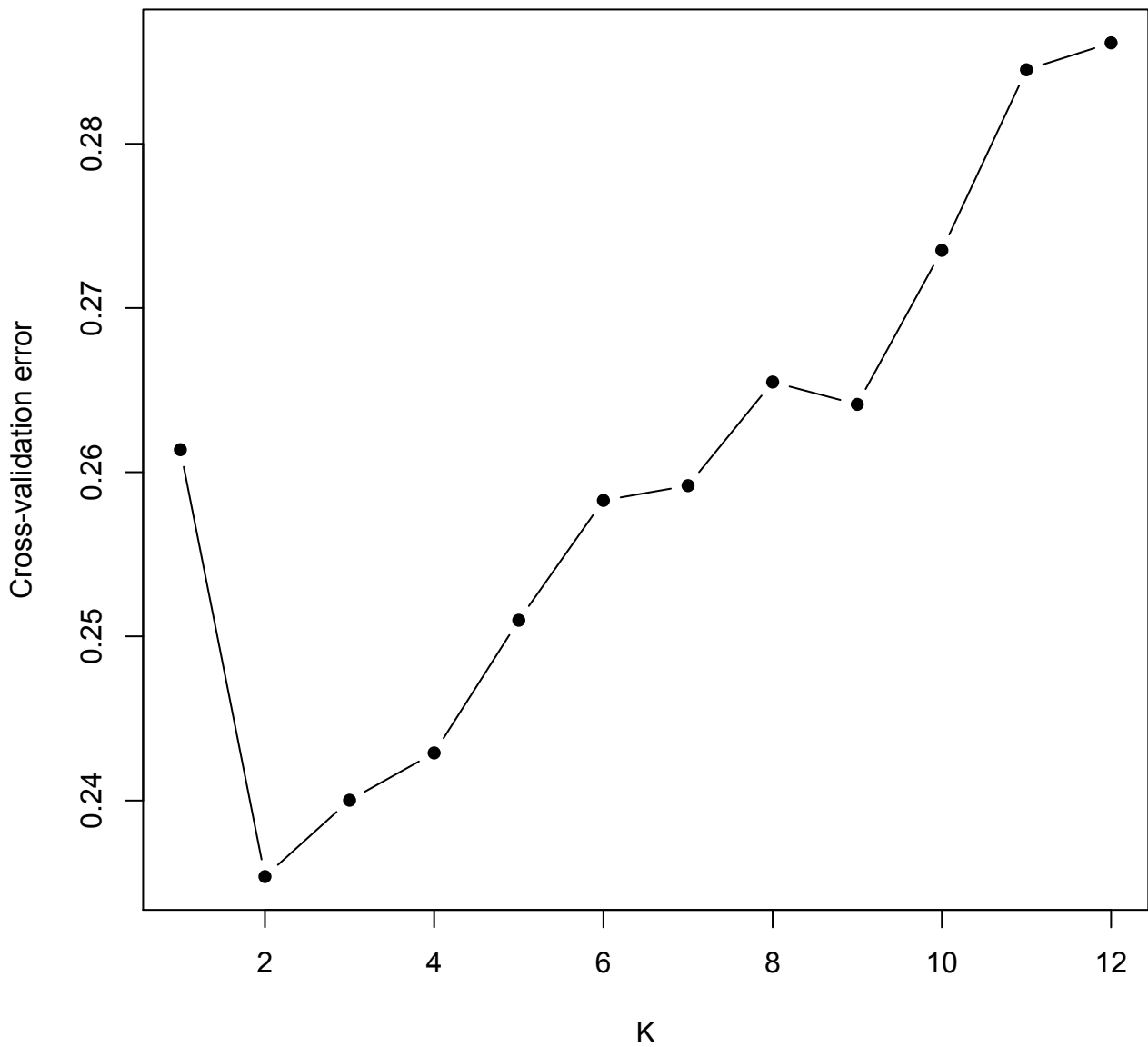

Supplement: Supplementary file 7 — Figure S4. Cross‐validation of results from ADMIXTURE for K = 1 – 12. [file EVA-9-546-s007.pdf]

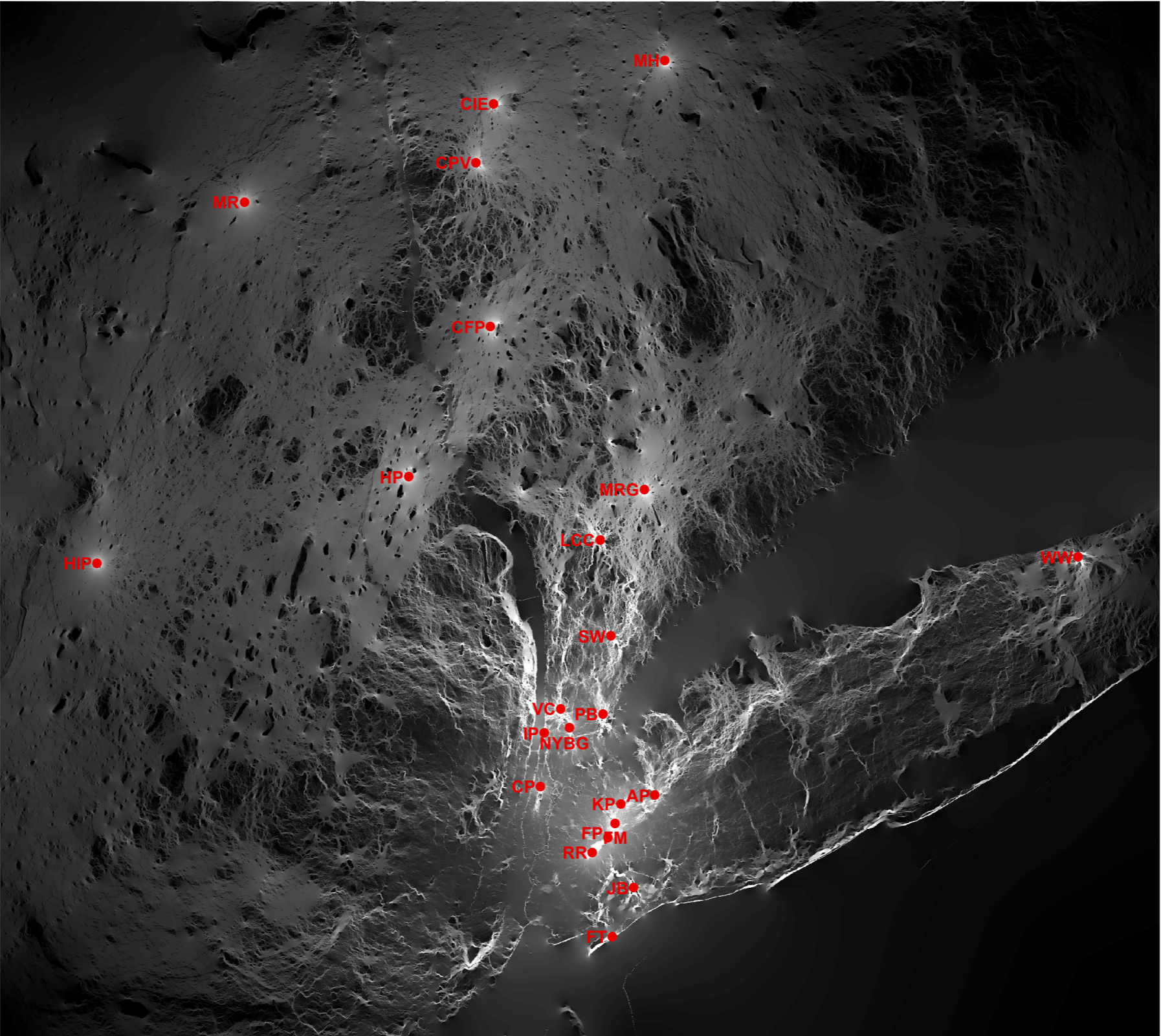

Supplement: Supplementary file 8 — Figure S5. Cumulative current map from isolation by resistance (IBR) modeling in Circuitscape. Lighter areas represent landscape cells with higher cumulative predicted current (i.e., higher movement). [file EVA-9-546-s008.pdf]
